# A Case of Intussusception Caused by Small Intestinal Metastasis From Primary Lung Cancer and Literature Review

**DOI:** 10.1002/ccr3.73192

**Published:** 2026-07-22

**Authors:** Pei Xin, Linghong Kong, Shuangqi Wang, Wei Jinping, Yanchao Sun, Zongbei Li, Yongzhe Wu, Huazhi Li

**Affiliations:** ^1^ Department of General Surgery Chuiyangliu Hospital Affiliated to Tsinghua University Beijing China; ^2^ Pathology Department Chuiyangliu Hospital Affiliated to Tsinghua University Beijing China; ^3^ Infection Control Department Chuiyangliu Hospital Affiliated to Tsinghua University Beijing China

**Keywords:** gastrointestinal (GI) metastasis, intestinal intussusception, lung cancer, non‐small cell lung cancer (NSCLC), small intestinal metastasis

## Abstract

Lung cancer metastasis to the digestive tract is not common, and the clinical manifestations are diverse. Now we report a case of intussusception caused by small intestine metastasis from lung cancer.

## Key Clinical Message

Lung cancer metastasis to the small intestine is extremely rare and presents with diverse clinical symptoms. It is usually diagnosed only when patients present with symptoms such as digestive tract bleeding, obstruction, perforation, or intussusception. Early diagnosis is extremely important for treatment.

## 1 Introduction

Lung cancer is one of the common malignant tumors with higher incidence and mortality, approximately 50% of cases have already metastasized by the time of diagnosis [1]. Lung cancer is classified into non‐small cell lung cancer (NSCLC) and small cell lung cancer (SCLC) based on histopathological characteristics. NSCLC constitutes over 85% of all lung malignancies, encompassing adenocarcinoma, squamous cell carcinoma, and large cell carcinoma, as well as rare subtypes including carcinoid tumor and sarcomatoid carcinoma [2, 3]. For NSCLC, the most common sites of metastasis are the brain (47%), bones (36%), liver (22%), adrenal glands (15%), thoracic cavity (11%), and distant lymph nodes (10%). Metastases to other organs are infrequent, accounting for less than 5% of cases [4, 5]. The occurrence of gastrointestinal (GI) metastasis from lung cancer has been reported to be 0.3%–1.77%. The sites where lung cancer metastasizes to GI include the stomach, duodenum, bile duct, colon, rectum, and small intestine; sometimes, metastasis occurs in multiple sites simultaneously [6]. Among them, the most common sites are the small intestine, colon, and stomach, mainly manifesting as obstruction, perforation, abdominal pain or distension, bleeding, constipation, vomiting, anemia [7, 8, 9]. Among NSCLC subtypes, squamous cell carcinoma (28.5%), adenocarcinoma (27.6%), and large cell carcinoma (20.9%) are the top three histological types with high propensity for distant metastasis [7]. It was observed that the jejunum and ileum are the most common sites of GI metastasis, followed by the duodenum [10, 11]. The main pathological type of metastatic tumors is poorly differentiated adenocarcinoma. Rare metastases indicate a poor prognosis. This article reports a lung cancer patient who had small intestine metastasis and suffered from intussusception.

## 2 Case History/Examination

The patient is a 66‐year‐old male. He was diagnosed with lung cancer 3 months ago. The lesion is located in the upper lobe of the left lung (Figure 1a). At the time of diagnosis, there were already lymph node metastases in the mediastinum and left pulmonary hilum. There were no surgical indications, and the patient refused chemotherapy and chose to receive treatment with traditional Chinese medicine.

**FIGURE 1:**
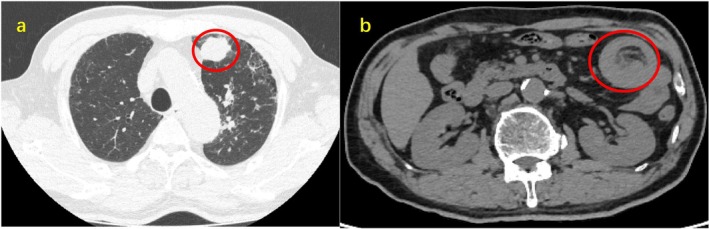
Imaging materials of the patient. (a) The chest CT scan showed a mass in the upper lobe of the left lung, along with metastasis to the left hilar lymph nodes and the mediastinal lymph nodes. (b) The abdominal CT scan showed “sleeve sign” formation in the middle abdomen, with gas accumulation and dilation in the proximal part of the intestinal tract. It was considered intestine intussusception.

During the treatment period, the patient experienced loss of appetite, accompanied by symptoms such as nausea, vomiting, and upper abdominal pain. Chest CT showed that the lung lesions had increased in size, and the lymph nodes in the left pulmonary hilum and mediastinum had significantly increased in number and size compared to before. A new nodule was also detected in the right lower lobe of the lung, which is considered to be a metastatic tumor. Abdominal CT showed intestinal intussusception of the middle abdomen (Figure 1b).

## 3 Differential Diagnosis, Investigations and Treatment

The patient was admitted to the department of geriatrics and a consultation with the general surgery department was requested. Physical examination revealed an abdominal mass in the middle abdomen, with poor mobility and tenderness. The surgeon suspected it to be intestinal intussusception. We suggested the patient an emergency laparoscopic exploration surgery, but the patient's family refused. Then we provided the patient conservative treatment, such as fasting and fluid infusion, GI decompression and relaxing the bowels. After 3 days, abdominal symptoms have not improved and abdominal CT showed intestinal intussusception was the same as before. We performed an emergency laparoscopic exploration surgery. The intestinal intussusception was confirmed at approximately 30 cm from the beginning of the jejunum, with dilatation of intestine and the color of the tube remaining normal. Then we transferred to laparotomy and found a mass in the mesentery of the intussuscepted intestines with a diameter of 2 cm (Figure 2a,b). The intussuscepted intestines were manually repositioned, a 5 cm tumor could be touched in the small intestine, which had hard texture and caused narrow intestinal cavity (Figure 2c). The mesenteric mass was removed, the small intestinal mesentery and intestinal tube were separated and cut at 10 cm proximal and distal to the small intestinal tumor (Figure 2d), and termino‐terminal anastomosis was performed. The patient was transferred to the ICU for the following treatment and then returned to the general surgery ward the day after surgery.

**FIGURE 2:**
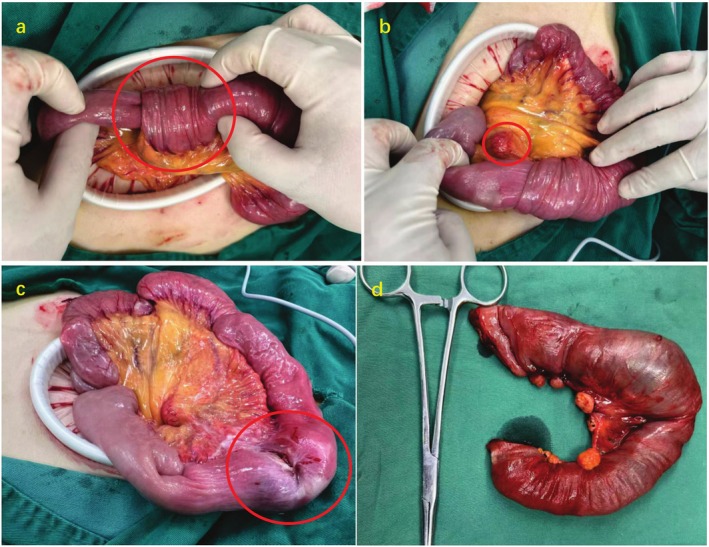
Abdominal surgery of the patient. (a) The intestinal intussusception was observed in the surgery. (b) A tumor nodule was observed in the mesentery corresponding to the area of intussusception. (c) The intussuscepted intestines were manually repositioned; a 5 cm tumor could be touched in the small intestine, which had a hard texture and caused a narrow intestinal cavity. (d) The small intestinal mesentery and intestinal tube were separated and cut at 10 cm proximal and distal to the small intestinal tumor.

The postoperative pathological diagnosis was dysdifferentiated malignant tumors with necrosis and was considered metastatic sarcomatoid carcinoma (Figure 3a). The tumor size was 6.5 × 4 × 2.5 cm, the tumor invaded the entire layer of the intestinal wall, with vascular tumor thrombus, no tumor cells were observed at the surgical resection margin. Tumor nodules were detected in the adipose tissue. The Immunohistochemical (IHC) staining was positive for CK, Vimentin, EMA, TTF‐1 (Figure 3b) and negative for CK5, CK7, LCA, CgA, P40, P63, CR, Napsin‐A. The final diagnosis is metastatic small intestinal sarcomatoid carcinoma, originating from the lung.

**FIGURE 3:**
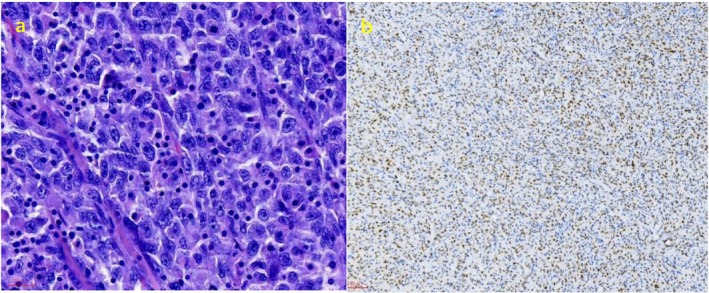
Pathological results. (a) Postoperative pathological findings, combined with clinical history and immunohistochemical results, are consistent with small intestinal metastasis of pulmonary sarcomatoid carcinoma. (b) HE ×200, Immunohistochemistry (TTF‐1) ×200.

## 4 Conclusion and Results (Outcome and Follow‐Up)

The patient recovered well and resumed a normal diet gradually after the surgery. As the patient refused anti‐tumor therapy such as chemotherapy and only accepting supportive treatment. The lung tumor grew rapidly and caused respiratory failure, so that the patient died 33 days after the surgery.

## 5 Discussion

Half of the lung cancer patients have already had metastasis in other organs by the time of diagnosis; the rare site metastasis of lung cancer adds difficulties for diagnosis and treatment. In this case, we hadn't considered the differential diagnosis of intestinal intussusception caused by metastatic tumor until the laparotomy was performed and the IHC pathology confirmed that the tumors originated from lung cancer. We reviewed a large number of literature to study the occurrence of lung cancer metastasis to the small intestine and wrote this article to share our experience and provide more diagnostic ideas for clinicians.

According to the statistics from 3 retrospective studies, primary lung cancer metastasis to the small intestine occurs in 2.6%–10.7% [12, 13, 14]. However, only a small number of patients show clinical manifestations, indicating that the incidence of primary lung cancer metastasis to the small intestine is higher than what we previously thought. Before severe complications occur, it is difficult to detect the hidden small intestinal metastasis at an early stage. Abdominal CT has low sensitivity in detecting small intestinal tumors; some indirect findings may be more meaningful, for instance, intestinal intussusception [15], small bowel obstruction [16]. Intestinal metastasis represents an advanced manifestation of lung cancer and predicts a poor prognosis [13]. The median survival time of GI metastasis patients was 2.8 months (range, 0–108 months) [7]. Poor prognosis is primarily associated with late diagnosis, the malignant biological characteristics of lung cancer, surgical complications, and distant metastasis [17].

The histological feature of the small intestine is the abundance of lymphoid tissue, including a large number of T lymphocytes, providing a high level of protection for the intestinal wall. The main way of intestinal metastasis is considered to be through hematogenous metastasis via venae spinales or through mediastinal lymph nodes in the retroperitoneum and mesentery [10]. Previous studies have identified cancer cells within the lymphatic vessels of resected intestinal tissue, suggesting a potential route for lung cancer metastasis to the intestines [18, 19, 20].

Immunohistochemical (IHC) diagnosis aids in identifying the primary tumor origin. Immunochemical markers including TTF‐1, Napsin‐A, CK7, CDX2, CK20, Villin, P40, P63, and CK5 can help distinguish metastatic lung cancer from primary GI cancer [21, 22, 23]. Positive staining for P40, P63, and CK5 can be related to squamous cell carcinoma. TTF‐1 regulates gene expression in the thyroid, lungs, and diencephalon during embryogenesis and aids in identifying tissues of pulmonary origin [24, 25]. Napsin‐A is a type of active aspartic proteinase that resides within the cytoplasm of healthy lung tissue cells. Combined with TTF‐1, this biomarker plays a critical role in the differential diagnosis of primary lung carcinoma and other adenocarcinoma subtypes [26].

There is no standardized treatment regimen for lung cancer with GI metastasis. Chemotherapy remains the primary therapeutic strategy for metastatic NSCLC [27, 28]. According to Lee's research, prolonged survival was observed in individuals with gastric and/or duodenal metastatic lesions who were treated with supportive management without surgery [29]. Conversely, some studies reported that aggressive surgical treatment for NSCLC with gastric metastasis patients can obtain a better prognosis [30, 31]. In this case, the patient had lost the opportunity for surgery and refused chemotherapy treatment, which led to rapid disease progression and distant metastasis. The patient had a good postoperative recovery after abdominal surgery. However, due to the rapid growth of the primary tumor and the absence of anti‐tumor treatment, the patient died after a month of the surgery. Effective systemic anti‐tumor treatment may potentially prolong the survival period of the patient.

## Author Contributions

**Linghong Kong:** conceptualization, methodology, writing – original draft. **Yongzhe Wu:** conceptualization, methodology, writing – review and editing, supervision. **Wei Jinping:** methodology, software. **Yanchao Sun:** investigation, validation. **Huazhi Li:** conceptualization, methodology, supervision, writing – review and editing. **Shuangqi Wang:** conceptualization, methodology, software. **Pei Xin:** conceptualization, methodology, data curation, investigation, writing – original draft. **Zongbei Li:** methodology, funding acquisition.

## Funding

The authors have nothing to report.

## Consent

Written informed consent was obtained from the patient for publication of this case report and any accompanying images.

## Conflicts of Interest

The authors declare no conflicts of interest.

## Data Availability

The data are not publicly available due to privacy or ethical restrictions. The data that support the findings of this study are available on request from the corresponding author.
